# Predicting Sudden Sensorineural Hearing Loss Recovery with Patient-Personalized Seigel’s Criteria Using Machine Learning

**DOI:** 10.3390/diagnostics14121296

**Published:** 2024-06-19

**Authors:** Sanghyun Shon, Kanghyeon Lim, Minsu Chae, Hwamin Lee, June Choi

**Affiliations:** 1 Department of Biomedical Informatics, Korea University College of Medicine, Seoul 02708, Republic of Korea; sonatus@hotmail.com (S.S.); minsuchae@korea.ac.kr (M.C.); 2 Department of Otorhinolaryngology-Head and Neck Surgery, Korea University Ansan Hospital, Ansan-si 15355, Republic of Korea; kingsonl@hanmail.net

**Keywords:** idiopathic sudden sensorineural hearing loss, patient-specific hearing impairment, hearing recovery, prognosis, machine learning, SHapley Additive exPlanations value

## Abstract

Background: Accurate prognostic prediction is crucial for managing Idiopathic Sudden Sensorineural Hearing Loss (ISSHL). Previous studies developing ISSHL prognosis models often overlooked individual variability in hearing damage by relying on fixed frequency domains. This study aims to develop models predicting ISSHL prognosis one month after treatment, focusing on patient-specific hearing impairments. Methods: Patient-Personalized Seigel’s Criteria (PPSC) were developed considering patient-specific hearing impairment related to ISSHL criteria. We performed a statistical test to assess the shift in the recovery assessment when applying PPSC. The utilized dataset of 581 patients comprised demographic information, health records, laboratory testing, onset and treatment, and hearing levels. To reduce the model’s reliance on hearing level features, we used only the averages of hearing levels of the impaired frequencies. Then, model development, evaluation, and interpretation proceeded. Results: The chi-square test (*p*-value: 0.106) indicated that the shift in recovery assessment is not statistically significant. The soft-voting ensemble model was most effective, achieving an Area Under the Receiver Operating Characteristic Curve (AUROC) of 0.864 (95% CI: 0.801–0.927), with model interpretation based on the SHapley Additive exPlanations value. Conclusions: With PPSC, providing a hearing assessment comparable to traditional Seigel’s criteria, the developed models successfully predicted ISSHL recovery one month post-treatment by considering patient-specific impairments.

## 1. Introduction

According to the World Health Organization (WHO), hearing loss is a global cause of disability and ranks as the third major contributor to productivity reduction [1]. Hearing loss can be categorized into two types as follows: conductive hearing loss and sensorineural hearing loss. According to the differential diagnosis of hearing loss, sensorineural hearing loss (SNHL) is the most prevalent type, comprising the majority of all hearing loss cases [2]. SNHL is associated with abnormalities in the cochlea, auditory nerve, and central nervous system. The causes of SNHL include congenital issues, presbycusis, noise exposure, head trauma, Meniere’s disease, ototoxicity, systemic conditions such as meningitis and diabetes, vestibular schwannoma, autoimmune diseases, barotrauma, and perilymphatic fistula [3]. Sudden sensorineural hearing loss (SSNHL) is defined as sensorineural hearing loss of 30 dB or worse, occurring over at least three consecutive frequencies within 72 h [1]. Most cases of SSNHL are idiopathic, meaning no specific cause can be identified [4]. Despite the uncertainty in its pathogenesis, viral infections, cochlear membrane destruction, and vascular occlusion have been suggested as causes of Idiopathic Sudden Sensorineural Hearing Loss (ISSHL) [5,6]. To date, many studies have been conducted on the prognosis of hearing recovery in ISSHL, and the main poor prognostic factors identified are severe hearing loss, high-frequency hearing loss, recovery starting duration greater than 2 weeks, advanced age, history of vertigo, and late initiation of treatment [5,6,7]. Although there are variations among studies, severe hearing loss in this context refers to a PTA hearing threshold of 71 dB or higher, advanced age refers to an age over 60, and delayed initiation of treatment depends on the extent of the delay. Regarding the history of vertigo, cases involving Ménière’s disease or cerebrovascular diseases, which fall outside the definition of ISSNHL, are excluded.

Previous studies have developed machine learning models to accurately predict the prognosis of ISSHL [5,8,9,10,11]. The machine learning approach enables the analysis of extensive and intricate medical data, allowing for the extraction of concealed information that is often imperceptible to the human eye [9,12]. Through dataset analysis, these machine learning models can effectively distinguish between relevant and irrelevant variables [13]. This characteristic of machine learning enables accurate prognostic prediction. Therefore, machine learning methods are essential to prevent a decline in the quality of life and productivity of patients with ISSHL. In previous studies, various machine learning models were developed using clinical variables and targets indicating recovery from ISSHL. To determine this target, these studies consistently applied specific hearing frequency ranges—“0.5, 1, 2, and 3 kHz” or “0.5, 1, 2, and 4 kHz”—across all patients, assessing recovery according to Siegel’s criteria within these frequencies [9,10,11]. The level of hearing impairment is represented as the average hearing threshold across the defined frequency domains. Siegel’s criteria assess hearing recovery based on the recent average hearing threshold and its improvement. Subsequently, model development and performance optimization are conducted through feature selection and parameter tuning, respectively. The model with the best performance is selected, and variables with high importance for prognosis prediction are identified.

However, relying solely on these fixed-frequency domains to assess hearing recovery may overlook the nuances of patient-specific hearing impairment, potentially resulting in imprecise evaluations. This oversight is particularly critical for cases involving atypical hearing loss patterns, such as high-frequency (0.3, 0.4, and 0.8 kHz) or low-frequency (0.125, 0.25, and 0.5 kHz) losses, which may not align with the fixed-frequency domains used. Such discrepancies can lead to an under-representation of the patient’s hearing loss, thereby skewing recovery assessments and possibly overestimating therapeutic success. Therefore, for a more accurate evaluation of ISSHL recovery, it is imperative to consider the hearing-impaired frequency domains specific to each patient’s condition. Additionally, there was an excessive impact of hearing threshold on the ISSHL prognosis prediction of the machine learning model in previous studies [5,9,10,11]. The hearing thresholds of various hearing frequency domains, also known as pure tone audiometry (PTA) records, contribute to the prediction of machine learning models with high importance. The hearing threshold of each frequency domain and the average value of the hearing threshold have been included as model variables. For example, in a recent study [9], hearing thresholds of 0.125, 0.25, 0.5, 1, 2, 3, 4, and 8 kHz; average hearing thresholds at low, mid, and high frequencies in the affected ear; and PTA records in the unaffected ear were engaged in ISSHL prognosis prediction. This redundancy in PTA variables reduces the efficiency of the model owing to the computational cost and high dimensionality of the dataset.

Although one study successfully predicted the prognosis of ISSHL based on hearing-impaired frequencies using artificial intelligence methods, further clarification of how the model functions is necessary [8]. The study reported the rank of influential variables in the model, but it did not disclose whether the impact of the key variables on the ISSHL prognosis prediction of the model was positive or negative [8]. Utilizing model-explanation techniques is essential for clarifying both the significance of the variables and the impact of the model’s influential variables on ISSHL prognosis prediction. This strategy ensures that the predictions of ISSHL prognosis, which are tailored to individual hearing impairments, are clinically understandable. Additionally, the difference in recovery assessment between traditional Siegel’s criteria and Seigel’s criteria focusing on hearing impairment is not known.

The primary objective of this study is to develop machine learning models for predicting the prognosis of patients with ISSHL one month after treatment, focusing on hearing-impaired frequency domains. A hearing-impaired frequency range is defined as at least three consecutive frequency ranges, each with a hearing threshold of 30 dB or more, aligned with the definition of ISSHL. Only the average PTA values of the affected ear, unaffected ear, and its categorical variables are included as hearing threshold variables so that the minimal set of hearing threshold variables are utilized. Subsequently, machine learning models were constructed based on clinical variables and hearing assessments. We elucidated the effects of clinical variables on the prediction of the model. We reviewed previous studies on ISSHL prognostic factors to explain the effects of the variables clinically. In addition, we statistically tested the distribution shift in the recovery assessment when changing the application from traditional Seigel’s criteria to Patient-Personalized Seigel’s criteria.

## 2. Materials

### 2.1. Data Collection and Study Population

**Figure 1:**
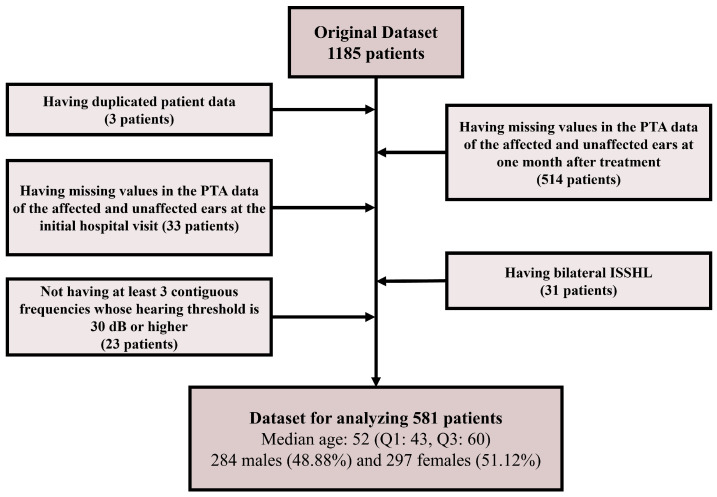
Procedural flow of patient exclusion. The content of the box with darker color indicates the dataset. And the content of the box with lighter color indicates the procedure of the patient exclusion. PTA: pure tone audiometry. dB: decibels. ISSHL: Idiopathic Sudden Sensorineural Hearing Loss.

We retrospectively reviewed the clinical records of 1185 patients with ISSHL who were admitted to the Department of Otorhinolaryngology-Head and Neck Surgery of Korea University Ansan Hospital between December 2009 and November 2022. All patients had sudden-onset idiopathic sensorineural hearing loss, defined as a hearing loss of ≥30 dB over 3 contiguous frequencies occurring within 3 days. Each participant underwent PTA following a medical interview and an otologic physical examination conducted by a board-certified otolaryngologist. The medical interview assessed the patient’s medical history, including conditions such as diabetes, hypertension, and myocardial infarction, and evaluated the presence of dizziness and tinnitus. Dizziness was evaluated solely as vertigo, characterized by a sensation of motion or spinning. Tinnitus was assessed as subjective tinnitus, defined as the perception of sound in the absence of an external stimulus, heard only by the patient. For PTA records, the hearing thresholds for all participants were assessed using an AC-40 audiometer, either the GSI 61 model from Grason-Stadler (Eden Prairie, MN, USA) or the Madsen Astera2 from Natus Medical (Taastrup, Denmark). The assessments took place in a soundproof booth, adhering to the clinical standards set by the International Organization for Standardization (ISO) [14]. Both air and bone conduction thresholds were measured at the standard frequencies of 0.125, 0.25, 0.5, 1, 2, 3, 4, and 8 kHz for both ears. To differentiate retrocochlear lesions, we performed auditory brainstem response (ABR) tests and/or brain magnetic resonance imaging on all patients. Patients with chronic otitis media, inner ear abnormalities on magnetic resonance imaging, or a history of surgery in the affected ear were excluded. All patients received treatment with either systemic steroids (e.g., methylprednisolone 64 mg orally for 4 days or dexamethasone 5 mg intravenously three times daily for 4 days. In both cases, methylprednisolone was started from the 5th day at 48 mg and tapered gradually every other day over 8 days), intratympanic dexamethasone injections (ITDIs) (1–4 times), or both. Since concurrent use of systemic steroids and ITDI has been reported as the most effective treatment for patients with ISSHL, concurrent therapy was recommended for all patients. However, in patients with contraindications to systemic steroid therapy, ITDI alone was administered, and in patients who refused ITDI, only systemic steroid therapy was administered. We examined the medical records of 581 patients to develop machine learning models. For data preparation, some patients were excluded sequentially by the following criteria: (1) the patient’s data were the same as another patient’s data (3 patients), (2) missing data existed in the patient’s PTA record measured one month after treatment (514 patients), (3) missing data existed in the patient’s PTA records of the affected ear and unaffected ear measured at the initial hospital visit (33 patients), (4) the patient had bilateral ISSHL (31 patients), and (5) the patient’s initial PTA record of the affected ear did not include at least three contiguous frequency domains, each with a hearing threshold of 30 dB or higher (23 patients). The procedure for patient exclusion is illustrated in Figure 1. Regarding the included 581 patients, the median age was 52 (Q1: 43, Q3: 60) and the gender distribution was 284 males (48.88%) and 297 females (51.12%). This study was approved by the Ethics Committee of our hospital (IRB No. 2022AS0088), which waived the requirement for informed consent because of the retrospective nature of this study. All methods were performed in accordance with the relevant guidelines and regulations.

### 2.2. Data Variables and Minimizing PTA Variables

In this study, 50 variables were selected to develop machine learning models. These variables were categorized into democratic information, health records, laboratory testing, onset and treatment, and PTA records. The original dataset contained 160 variables including patient ID variables. Initially, we excluded 71 variables that had more than half of their records missing, as well as two speech discrimination score variables with missing ratios of approximately 45%. Inaccurately imputed values for speech discrimination scores could potentially mislead the model in learning the patterns necessary for accurate prognosis prediction. Second, only variables related to the average hearing threshold of the hearing-impaired frequency range were included, and the detailed PTA variables of the affected and unaffected ears were excluded to minimize the number of PTA variables. These excluded PTA-related variables of the affected and unaffected ears included 0.125, 0.25, 0.5, 1, 2, 3, 4, and 8 kHz; the average hearing threshold at the 0.5, 1, 2, and 3 kHz frequency domains; its category features; and the average PTA values for low-frequency domains (0.125, 0.25, and 0.5 kHz), middle-frequency domains (1 and 2 kHz), and high-frequency domains (3, 4, and 8 kHz).

**Figure 2:**
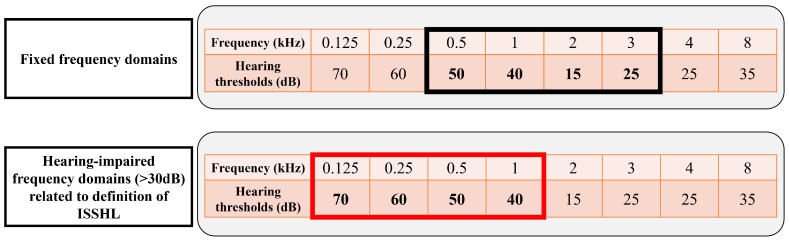
Example of the frequency domains of patient-specific hearing impairment. The fixed-frequency domains (0.5 to 3 kHz) are indicated in the black bold box, while those related to the definition of ISSHL (0.125 to 1 kHz) are indicated in the red bold box. In this study, the hearing-impaired frequency domains related to the definition of ISSHL are considered for hearing recovery assessment. Although the hearing threshold is over 30 dB, we did not include 8 kHz in the considered frequency domains for recovery assessment because it is a single frequency rather than at least three consecutive frequency domains and it is separate from the consecutive frequency range (0.125 to 1 kHz). ISSHL: Idiopathic Sudden Sensorineural Hearing Loss.

To calculate the average hearing threshold of the hearing-impaired frequency range, we initially defined the hearing-impaired frequency range. This frequency range consisted of at least three contiguous frequency domains, each with a hearing threshold of 30 dB or greater. In other words, we focused on the frequency domains that are directly related to the definition of ISSHL onset instead of considering all hearing-impaired frequency domains. This frequency range represents patient-specific hearing loss and serves as the basis for determining ISSHL onset. A description of the frequency range of patient-specific hearing loss is shown in Figure 2 along with an example. Hearing-impaired frequency domains are identified in the PTA record of the affected ear at the initial hospital visit. These frequency domains were applied to the PTA records of affected and unaffected ears to calculate the average hearing threshold at the initial hospital visit. Further, their categorized features were made via the following criteria: the range lower than 40 dB was defined as hearing level 1 (mild), the range from 40 dB to 60 dB as hearing level 2 (moderate), the range from 60 dB to 80 dB as hearing level 3 (severe), the range from 80 dB to 100 dB as hearing level 4 (profound), and the range more than 100 dB as hearing level 5 (deaf).

Three categorical variables were encoded into binary variables as follows: (1) smoking status feature (non-smoker, smoker, smoking post-cessation) was encoded into two binary variables (smoking status and smoking post-cessation); (2) audiogram type of initial PTA record, which included five types (ascending, U-shaped, descending, flat, and deaf), was encoded into five binary variables; and (3) treatment-type features (systemic steroids, intratympanic dexamethasone injection, and the combination of both) was encoded into three binary variables. In addition, a feature related to the length of the hearing-impaired frequency domains in the affected ear’s initial PTA record was added, whereas the prothrombin time percentage feature was excluded. The affected consecutive frequency domains can cover part or all the measured frequency domains, resulting in variations in the lengths of the affected consecutive frequency domains among patients. The prothrombin time percentage feature shares the same attributes as prothrombin time (in seconds). Thus, the dataset retained 50 features. A detailed list of the features used in this study is presented in Table 1.

The remaining features comprising the utilized dataset included the following. The democratic information category consisted of age, height, weight, and gender. The health records category consisted of the body mass index, extent of smoking (packs/year), systolic blood pressure, diastolic blood pressure, smoking, smoking post-cessation status, and eight variables regarding presence of disease including (1) hypertension, (2) diabetes, (3) stroke, (4) dizziness, (5) tinnitus, (6) hyperlipidemia, (7) chronic kidney disease, (8) myocardial infarction or angina. The laboratory testing category consisted of total cholesterol, low-density lipoprotein (LDL), triacylglycerol, hemoglobin, blood urea nitrogen (BUN), creatine (Cr), white blood cell count, neutrophil count, lymphocyte count, neutrophil-lymphocyte ratio, platelet count, prothrombin time, and activated partial thromboplastin time. Onset and treatment consisted of the duration between the onset of ISSHL and initial treatment, the duration between the onset of ISSHL and initial intratympanic dexamethasone injection (ITDI) treatment, hospitalization, affected side, categorized variables of the duration between the onset of ISSHL and initial ITDI treatment, onset month of ISSHL, length of the hearing-impaired frequency domain, three variables of steroid treatment type including systemic steroid, ITDI, and the combined method of systemic steroid and ITDI. PTA records consisted of the PTA average of the affected frequency domains in the affected and unaffected ears, categorized variables of the PTA average of the affected frequency domains in the affected and unaffected ears, and five variables of audiogram type regarding the initial PTA record including ascending, U-shaped, descending, flat, and deaf.

## 3. Methods

### 3.1. Recovery Assessment by the Newly Developed Patient-Personalized Seigel’s Criteria

**Figure 3:**
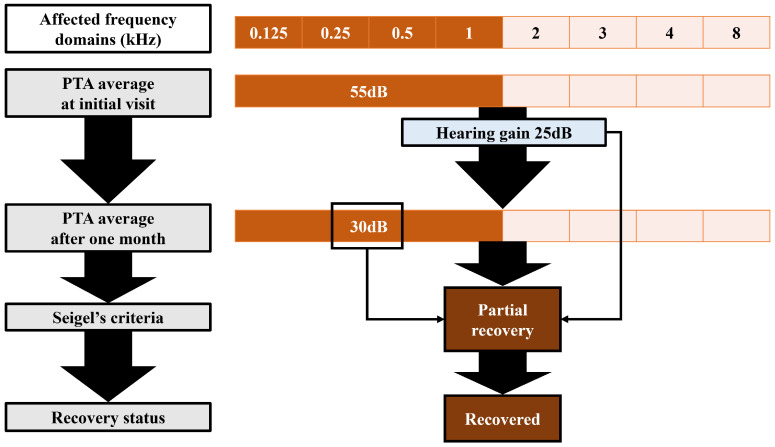
Example evaluation of the recovery of ISSHL by Patient-Personalized Seigel’s criteria. In this case, the affected frequency domains encompass 0.125, 0.25, 0.5, and 1 kHz, represented as light brown regions. The average PTA value of this specific frequency region is calculated, along with the PTA value recorded after one month of treatment. Consequently, the improvement in the PTA average amounts to 25 dB, with the PTA average after one month of treatment reaching 30 dB. According to Patient-Personalized Seigel’s criteria, this case is assessed as partial recovery. PTA: pure tone audiometry.

The recovery status of ISSHL, which is the target of the machine learning model, was determined using Patient-Personalized Siegel’s criteria. The recent hearing level and hearing improvement in the average hearing threshold in the hearing-impaired frequency domains were calculated. Siegel’s criteria [15] were then applied to assess recovery from ISSHL after one month of treatment, referred to as Patient-Personalized Siegel’s criteria. If the average PTA value of the affected frequency domains was 25 dB or lower after one month of treatment, the assessment indicated complete recovery. If the hearing gain was >15 dB and the average PTA value was >25 dB but within 45 dB, the assessment indicated a partial recovery. If the hearing gain was >15 dB and the average PTA value was >45 dB, but within 75 dB, the assessment indicated a slight recovery. Finally, no recovery was observed if the difference was <15 dB or the average PTA value was >75 dB. Complete and partial recovery were considered recovered statuses, whereas slight recovery and no recovery were considered unrecovered statuses. An example of the ISSHL recovery assessment is shown in Figure 3. Additionally, we compared the distribution of recovered and unrecovered patients when Seigel’s criteria were applied to the 0.5, 1, 2, and 3 kHz frequency domains and hearing-impaired frequency domains.

### 3.2. Statistical Analysis to Investigate Clinical Characteristics of ISSHL Patients

After evaluating the recovery results, a two-sided statistical analysis was conducted at a significance level of 0.05 to determine differences between patients who recovered from ISSHL and those who did not. We conducted a Shapiro–Wilk test to assess the normality of the distribution of continuous variables. For continuous variables that followed a normal distribution, data were summarized as means and standard deviations, and an independent sample t-test was used to compare the recovery and non-recovery groups. Continuous variables that did not follow a normal distribution were summarized using medians and interquartile ranges. The Mann–Whitney U test was used to compare two groups for not normally distributed features. Categorical variables were presented as patient counts and percentages, and the two groups were compared using chi-square or Fisher’s exact tests. Statistical analyses comparing two groups were conducted using IBM SPSS Statistics version 26.

### 3.3. Machine Learning Models

#### 3.3.1. Logistic Regression

A logistic regression model is a binary classifier that can determine the recovery or non-recovery of a disease and learn the relationship between variables and the recovery status of the disease [16]. This model assumes that the log of the odds is linearly related to the variables. Logistic regression is a linear regression model in which the output is log odds. Probability is computed based on the trained logistic regression model.

#### 3.3.2. Decision Tree

A decision tree model is hierarchically organized into nodes [17]. Classification commences with data at the root node, where data undergo successive partitioning into two subgroups guided by decision functions housed within internal nodes. This process is repeated at each internal node, and the data division culminates at the leaf nodes, ultimately leading to the determination of the final classification results. As the classification progresses from the root node to the leaf nodes, the class purity is enhanced, and the classification process ceases upon meeting the predefined termination criteria.

Instead of adjusting the minimal sample size and maximal depth, we applied cost complexity pruning to introduce the penalty for tree size [18,19]. The cost complexity pruning algorithm computes the total cost of a decision tree by combining the misclassification cost (error) and a complexity penalty proportional to the number of leaves in the tree [18,19]. The cost complexity pruning method prunes decision trees to minimize a cost complexity $R_{\alpha }\left(T\right)$ [19]. The formula is as follows, where $R\left(T\right)$ represents the misclassification cost of the tree T, α represents the complexity parameter, and $\left|T\right|$ represents the number of leaves.

$$R_{\alpha }\left(T\right)=R\left(T\right)+\alpha \times \left|T\right|$$

By controlling α complexity parameter, the extent of pruning of the tree can be adjusted [19]. Increasing the value of α prunes the tree more, removing branches that contribute little to reducing the misclassification cost relative to their complexity penalty [19]. This process could simplify the tree and help prevent overfitting [19]. Conversely, decreasing the value of α allows for a more complex tree that may capture more intricate patterns, but there is a risk of overfitting [19]. In this study, the α complexity parameter was adjusted by controlling the “cpp_alpha” value of scikit-learn API.

#### 3.3.3. Support Vector Machine

The Support Vector Machine (SVM) is a binary classification model designed to discover the most suitable hyperplane for classification in a high-dimensional space [20]. A linear SVM employs either a maximum-margin approach or soft-margin approach for classification. A maximum-margin SVM classifier is trained to maximize the distance between the classification hyperplane and the nearest data points to that hyperplane. By contrast, a soft-margin SVM classifier is trained to tolerate misclassifications caused by noise and outliers commonly found in real-world datasets. Nonlinear SVM models use diverse kernel functions to transform data into high-dimensional feature spaces.

#### 3.3.4. Random Forest

The Random Forest ensemble comprises numerous decision trees, with each tree trained on a distinct dataset derived through bootstrapping [21]. The final classification outcome is ascertained through a majority vote, wherein predictions from various decision trees are aggregated.

#### 3.3.5. Adaptive Boosting 

Adaptive Boosting (AdaBoost) instructs weak classifiers using a dataset that highlights the significance of certain elements, followed by an iterative reassessment of the importance of patient data grounded in classification errors [22]. This iterative procedure entails the successive training of weak classifiers on datasets with readjusted importance. With each new iteration, a fresh classifier is trained on the dataset. Upon the culmination of these iterations, the ultimate classification outcome is established through a weighted collective vote from each classifier.

#### 3.3.6. Extreme Gradient Boosting and the Light Gradient Boosting Model

Extreme gradient boosting (XGBoost) and the Light gradient boosting model (LGBM) consist of decision trees trained sequentially [23,24]. These trees are specifically designed to mitigate prediction errors stemming from their predecessors. In the XGBoost model, decision trees expand in a level-wise manner, whereas in the LGBM model, they expand leafwise. The XGBoost and LGBM models from the ‘xgboost’ (1.7.2 version) and ‘lightgbm’ (3.3.4 version) Python packages, respectively, are utilized in our study, as depicted in the Table S1.

#### 3.3.7. K-Nearest Neighbors

The K-Nearest Neighbor (KNN) model conducts non-parametric classification by utilizing a pre-existing dataset, eliminating the need for a distinct training procedure [25]. For a new observation, the model determines the k-nearest data points and assigns the class that appears most frequently among these data points as the classification result for the new observation [5,25].

#### 3.3.8. Soft-Voting Ensemble

A voting ensemble model comprises multiple base models, and the predictions of these models are integrated through a vote to derive the final classification outcome with the highest score [26]. Voting ensemble models primarily employ hard and soft voting methods. Hard voting combines the classification results of the base models through majority voting, where the class with the highest frequency is the final classification result. Soft voting averages the predicted probability results of the base models, and the class with the highest probability is considered the overall classification result.

### 3.4. Model Development Process

The methodology of this study encompassed three key phases including validation, test evaluation, and visualization of SHapley Additive exPlanations (SHAP) summary plots. Initially, the original dataset was partitioned into training and test datasets comprising 80% and 20% of the patients, respectively. Validation involved 5-fold stratified cross-validation [27] applied to the training dataset across ten different combinations. Each cross-validation cycle split the training dataset into an 80% sub-training dataset and a 20% validation dataset based on the number of patients in the training dataset. Data preprocessing was conducted using Multiple Imputation by Chained Equations (MICE) [28] and min–max scaling [29]. In our study, MICE was implemented in a round-robin fashion [30,31]. Each feature with missing values was imputed sequentially in a cyclic manner until the algorithm converged to stable estimates [30]. Initially, each feature selected for imputation was addressed in the specified order starting from features with the fewest missing values to those with the most [30]. Other missing feature values were initially filled with their median values [30]. A multivariate regression model then predicted the missing values for the selected feature [30]. This step was repeated for each feature, completing one cycle of the round-robin iterative process and producing updated imputed values distinct from the initial median imputations [30]. The process iterates through multiple rounds, using the previously estimated values until the imputed values converge across all features [30]. The maximum number of imputation iterations was set to 20. Following MICE imputation, integer-type variables were rounded off before applying min–max scaling. The models were then trained with the best parameters via hyperparameter tuning, and their performance was evaluated using the validation dataset. The overall performance metric was the average of all iterations within this process. The test dataset underwent a similar procedure in which it was subjected to MICE imputation and min–max scaling, followed by model training and performance evaluation. Model performance metrics included balanced accuracy, recall, precision, F1 score, and Area Under the Receiver Operating Characteristic Curve (AUROC) [32,33,34]. Balanced accuracy is the average of sensitivity (recall) and specificity, providing a balanced measure for imbalanced datasets [32]. Recall (sensitivity) is the proportion of actual positives correctly identified by the model [32]. Precision is the proportion of positive predictions that are correct [32]. The F1 score is the harmonic mean of precision and recall, providing a single measure of the model’s accuracy [32]. The ROC curve evaluates the performance of a binary classification model by plotting sensitivity against 1-specificity across various thresholds, illustrating the trade-off among these metrics [33,34]. The AUROC quantifies this performance as a single value, representing the area under the ROC curve [33,34]. A higher AUROC indicates better model performance, with a value closer to 1 being ideal [34]. The formulas for the metrics are as follows, where TP, FP, FN, and TN represent True Positive, False Positive, False Negative, and True Negative, and t represents the threshold values used to determine the ROC curve.

$$Balanced\;Accuracy=\frac{1}{2}\left(\frac{TP}{TP+FN}+\frac{TN}{TN+FP}\right)$$

$$Recall=\frac{TP}{TP+FN}$$

$$Precision=\frac{TP}{TP+FP}$$

$$F1\;Score=2\times \frac{Presicion\times Recall}{Presicion+Recall}$$

$$AUROC=\int_{0}^{1}ROC(t)dt$$

True Positive is the number of correctly identified positive instances, False Positive is the number of instances incorrectly identified as positive, False Negative is the number of instances incorrectly identified as negative, and True Negative is the number of correctly identified negative instances [32].

**Figure 4:**
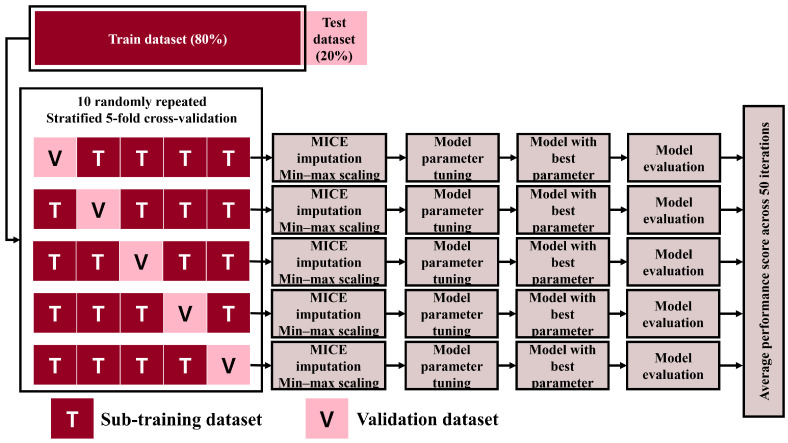
Overall procedure of the validation stage. MICE: Multiple Imputation by Chained Equations.

**Figure 5:**
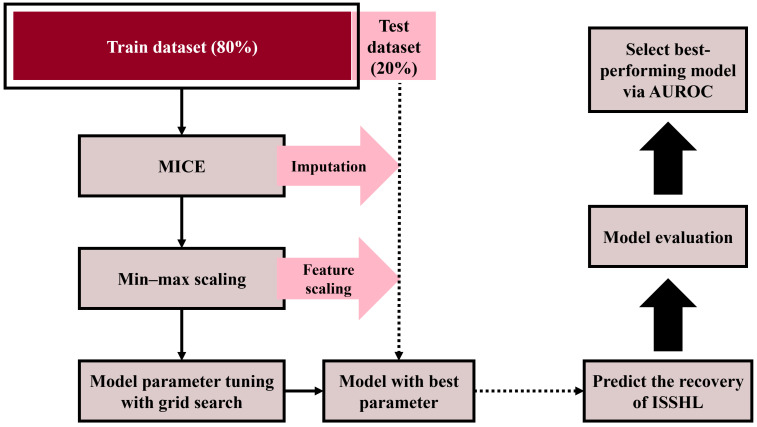
Overall procedure of the test evaluation stage. The entire dataset is split into the train dataset (80% of patients, wine box) and the test dataset (20% of patients, pink box). The train dataset undergoes data preprocessing and model training (black solid arrows), while the test dataset undergoes data preprocessing and ISSHL prediction (black dotted arrows). The pink arrow indicates the application of MICE imputation and min-max scaling aligned with the train dataset. Finally, model evaluation and selection of the best model with the highest AUROC are conducted (black bold arrows). AUROC: Area Under the Receiver Operating Characteristic Curve.

The model with the highest AUROC for the validation and test evaluations was selected as the best-performing model. Figure 4 and Figure 5 show the overall procedures for the validation and test evaluation stages, respectively.

**Figure 6:**
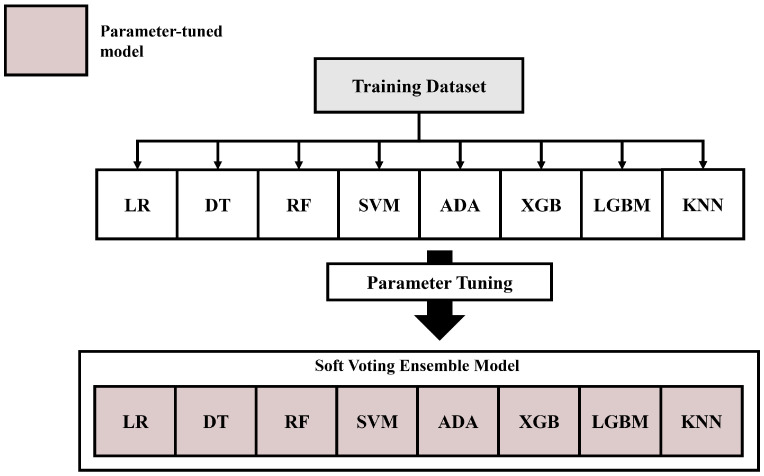
Construction of the soft-voting ensemble classifier. The performance of each of the consistent models is optimized through a grid-search method with the training dataset (gray box). The soft voting ensemble model consists of the models with an optimized parameter set and is trained with the training dataset. The models with non-tuned parameters are colored white, while those with tuned parameters are colored light brown. The abbreviations are as follows: LR: logistic regression, DT: decision tree, RF: Random Forest, SVM: Support Vector Machine, ADA: Adaptive Boosting (AdaBoost), XGB: Extreme Gradient Boosting (XGBoost), LGBM: Light Gradient Boost Model, KNN: K-Nearest Neighbor.

To optimize the AUROC score, a grid-search hyperparameter tuning algorithm [35] was employed based on 3-fold stratified cross-validation. Further, in Scikit-learn (1.2.2 version) and lightgbm (3.3.4 version) python package tools utilized in our study, the “class_weight” parameter of machine learning models such as logistic regression, decision tree, Random Forest, SVM, and LGBM were adjusted with “balanced,” ensuring each class’s weight was inversely proportional to its frequency [36,37]. This adjustment was implemented throughout this study. A soft voting ensemble model incorporating these optimized models was constructed to enhance the AUROC score. The construction of the ensemble model is depicted in Figure 6, and Table 2 lists the parameter settings and optimal parameter configurations for the entire training dataset.

### 3.5. SHAP Values

In this study, SHAP [38] values were computed using the outcomes of the top-performing model during the test evaluation phase. The significance of each variable was quantified by calculating the mean absolute SHAP values [38,39]. This process led to the identification of the 20 most influential variables. The SHAP summary plot graphically represents the relationships among these variables and the predictive outcomes of the model.

The SHAP summary plot for the best-performing model was used to elucidate the influence of the key variables on prognosis prediction. In the SHAP summary plot, the horizontal axis denotes the SHAP values, indicating the degree of impact of the variable on the model’s predictions. The vertical axis ranks the features based on their relative importance, with more critical features positioned higher than those with lower significance. This plot is an amalgamation of the individual dot plots for each variable, where each dot symbolizes specific patient data. In these dot plots, the color of each dot reflects the value of the corresponding variable. A transition towards red indicates an increase in the value of the variable, whereas a shift towards blue indicates a decrease. The color gradient serves as an intuitive indicator of the influence of the variable; red-hued dots at higher SHAP values suggest a positive contribution to the model’s prediction, whereas blue-hued dots imply a negative influence.

## 4. Results

### 4.1. Impact of Patient-Personalized Siegel’s Criteria on the Recovery Distribution

The application of traditional Siegel’s criteria, focusing on the 0.5, 1, 2, and 3 kHz frequency domains, identified 334 patients (57.5%) as non-recovery and 247 patients (42.5%) as recovery. However, when this study implemented Patient-Personalized Siegel’s criteria, which were tailored to each patient’s specific frequency domain impairments, there was a noticeable shift in the distribution as follows: 361 patients (62.1%) were categorized as non-recovery and 220 patients (37.9%) were classified as recovery. This implementation resulted in a change in the recovery status of 41 patients. Notably, 7 patients were reclassified from non-recovery to recovery, whereas 34 moved from recovery to non-recovery. To evaluate the statistical significance of the observed shift, we conducted a chi-square test using SPSS software version 26. The input table was structured with rows representing the method (traditional Seigel’s criteria and Patient-Personalized Seigel’s criteria) and columns representing the recovery state (recovered or non-recovered). Specifically, the table included 334 recovered and 247 non-recovered patients according to traditional Seigel’s criteria and 220 recovered and 361 non-recovered patients according to Patient-Personalized Seigel’s criteria. The chi-square test yielded a value of approximately 2.61, with a *p*-value of 0.106, indicating no statistically significant difference (*p* > 0.05).

### 4.2. Clinical Characteristics of ISSHL Patients according to Patient-Personalized Seigel’s Criteria

The distribution of age and gender in the non-recovery and recovery groups was as follows: In the non-recovery group, the median age was 55 (Q1: 47, Q3: 64), and there were 185 males (51.25%) and 176 females (48.75%). In the recovery group, the median age was 48 (Q1: 38.25, Q3: 57), and there were 99 males (45%) and 121 females (55%). The statistical comparison between the recovery and non-recovery groups, focusing on variables with significant differences (*p* < 0.05), is detailed in Table 3. The statistical comparison between the recovery and non-recovery groups on all variables is clarified in Table S2. The variables demonstrating significant disparities (*p* < 0.001) included age, blood urea nitrogen (BUN) level, PTA averages in hearing-impaired frequencies for both affected and unaffected ears, hypertension, dizziness, and various audiogram types (ascending, U-shaped, flat, and deaf). In general, the median values of the continuous variables were higher in the non-recovery group than in the recovery group. For categorical variables, apart from tinnitus and some audiogram types (ascending, U-shaped, and flat), higher percentages were observed in the non-recovery group.

In the demographic category, the age variable showed a significant difference between the recovery and non-recovery groups. The non-recovery group (median age 55, Q1: 47, Q3: 64) was older than the recovery group (median age 48, Q1: 38.25, Q3: 57).

In the health records category, significant differences were observed in the presence of hypertension, diabetes, myocardial infarction or angina, dizziness, and tinnitus between the recovery and non-recovery groups. The non-recovery group had higher frequencies of hypertension ((131 patients, 36.29%) vs. (46 patients, 20.91%), (number of patients, proportion of patients)), diabetes ((113 patients, 31.30%) vs. (47 patients, 21.36%)), myocardial infarction or angina ((21 patients, 5.82%) vs. (3 patients, 1.4%)), and dizziness ((152 patients, 42.11%) vs. (39 patients, 17.73%)). Conversely, tinnitus was less frequent in the non-recovery group ((230 patients, 63.71%) vs. (164 patients, 74.55%)).

In the laboratory testing category, triacylglycerol, blood urea nitrogen, and creatinine levels showed significant differences between the two groups. The non-recovery group had higher levels of triacylglycerol (99.00 mg/dL (66.50, 148.00) vs. 82.00 mg/dL (56.00, 132.00), median (Q1, Q3)), blood urea nitrogen (15.20 mg/dL (12.40, 19.58) vs. 13.60 mg/dL (11.50, 16.00)), and creatinine (0.88 mg/dL (0.71, 1.04) vs. 0.83 mg/dL (0.70, 0.98)) compared with the recovery group.

In the onset and treatment category, significant differences were found in the duration between onset and ITDI treatment, the categorization of this duration, and the length of the affected frequency range. The non-recovery group had a longer duration between onset and treatment (6.00 days (3.00, 16.00) vs. 5.00 days (2.00, 8.50), median (Q1, Q3)). Most patients in the non-recovery group received treatment after 13 days or more from symptom onset (73 patients, 20.22%), while most patients in the recovery group received it within 3 days (51 patients, 23.18%). The non-recovery group also had a higher frequency of the full-length affected frequency range (272 patients, 75.34% vs. 131 patients, 59.55%).

In the PTA records category, significant differences were observed in the PTA average of affected and unaffected ears, categorized severity levels, and audiogram types. Patients in the non-recovery group had more severe hearing loss in the affected ear (75.63 dB (56.77, 98.44) vs. 61.25 dB (48.33, 77.34)). Moderate hearing loss was most frequent in the recovery group (90 patients, 40.91%), while profound hearing loss was most frequent in the non-recovery group (119 patients, 32.96%). For the unaffected ear, the non-recovery group had a higher average hearing threshold (23.13 dB (15.00, 36.25) vs. 16.88 dB (10.83, 23.59)). The mild category was the most dominant category in both the recovery and non-recovery groups. But, in detail, more severe categories than mild categories were more frequent in the non-recovery group (79 patients, 21.88%) compared with the recovery group (13 patients, 5.91%). Lastly, the presence of ascending (35 patients, 9.69% vs. 47 patients, 21.36%), U-shaped (17 patients, 4.71% vs. 28 patients, 12.72%), and flat (81 patients, 22.44% vs. 83 patients, 37.73%) audiogram types were more frequent in the recovery group. Conversely, descending (119 patients, 32.96% vs. 53 patients, 24.09%) and deaf (109 patients, 30.19% vs. 9 patients, 4.09%) audiogram types were more frequent in the non-recovery group.

Additionally, patients treated solely with systemic steroids exhibited a lower degree of hearing loss compared with those treated with both systemic steroids and ITDI. However, this difference was not statistically significant according to the Mann–Whitney U test (median PTA (Q1, Q3): 65.31 (50.00, 86.72) dB vs. 72.50 (53.75, 91.25) dB, *p* = 0.066).

### 4.3. Model Performance and Key Variables

The soft voting classifier emerged as the most effective model, achieving the highest AUROC and precision scores among all the models tested in both validation and test evaluations. During validation, the performance metrics were as follows: AUROC, 0.775 (95% CI, 0.659–0.887); balanced accuracy, 0.686; recall, 0.597; precision, 0.620; and F1 score, 0.605. In the test evaluation phase, the model demonstrated an AUROC of 0.864 (95% CI, 0.801–0.927), balanced accuracy of 0.772, recall of 0.750, precision of 0.688, and F1 score of 0.717.

In addition, the logistic regression and Support Vector Machine models outperformed the soft voting classifier in terms of balanced accuracy, recall, and F1 scores. The detailed performance metrics for the validation and test evaluations are presented in Table 4 and Table 5, respectively.

**Figure 7:**
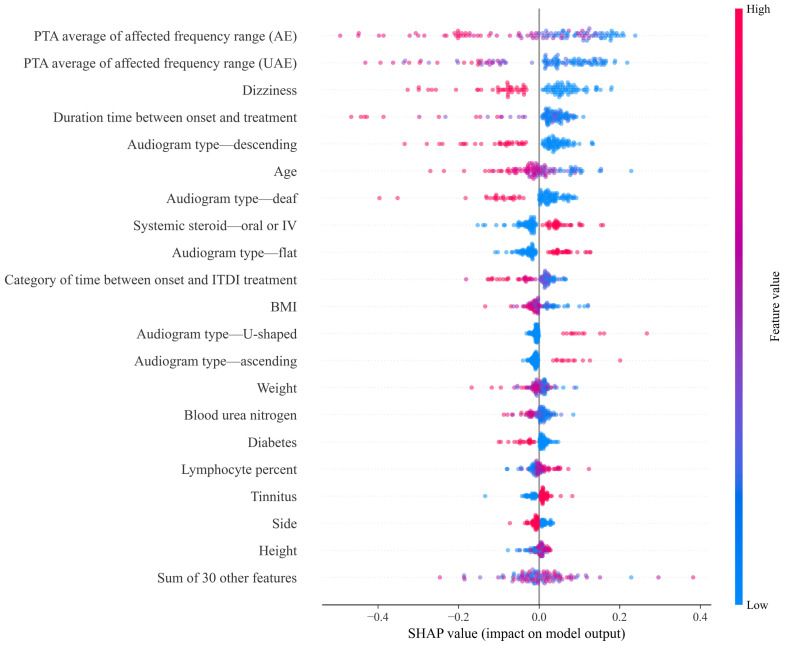
SHAP summary plot of the soft voting ensemble that depicts the relationship among the top 20 variables and the predicted outcome. AE: affected ear, UAE: unaffected ear, IV: intravenous injection, ITDI: initial intratympanic dexamethasone injection, BMI: body mass index.

Figure 7 shows the impact of the top 20 most influential variables on the recovery prediction of the soft voting classifier. The five variables with the most significant influence included the average PTA of the affected frequency in both the affected and unaffected ears, the time elapsed between the onset of symptoms and the initiation of treatment, the presence of dizziness, and the presence of a descending audiogram. Notably, all these variables negatively affected the prediction of recovery, indicating that higher values or the presence of these factors may be associated with a decreased likelihood of recovery.

## 5. Discussion

### 5.1. Impact of Applying Patient-Personalized Siegel’s Criteria on the Recovery Assessment

**Figure 8:**
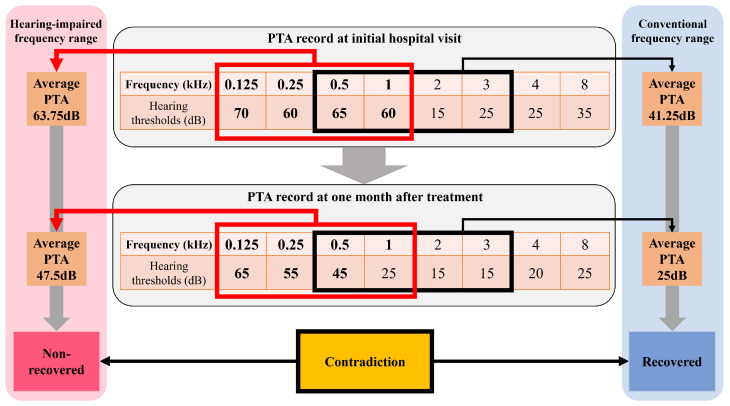
Example of a contradiction in recovery assessment of ISSHL in one patient. The conventional frequency range for recovery assessment is 0.5 to 3 kHz (black bold box and arrow). The hearing-impaired frequency range is 0.125 to 1 kHz (red bold box and arrow). In the hearing-impaired frequency range, their individual frequency domains and hearing thresholds are emphasized with bold style except the hearing threshold of 1 kHz domain after one month treatment which is lower than 30 dB. After one month of treatment, the patient is determined to have recovered according to Seigel’s criteria considering the conventional frequency range (light blue rounded square). However, the patient is determined to be non-recovered according to the Seigel’s criteria considering the hearing-impaired frequency range (light red rounded square). PTA: pure tone audiometry, dB: decibel.

The Patient-Personalized Siegel criteria led to notable changes in ISSHL recovery classification in 41 patients (7%). Specifically, 7 patients were reclassified from non-recovery to recovery, and 34 underwent a transition from recovery to non-recovery. This shift underscores the importance of considering patient-specific impaired frequency ranges in hearing assessments. Figure 8 shows how recovery status can differ based on the frequency range used for the assessment. In the example shown in Figure 8, the patient-specific hearing impairment is 0.125, 0.25, 0.5, and 1 kHz, and the average hearing threshold is 63.75 dB in the initial record and 47.5 dB at one month after treatment. With 0.5, 1, 2, and 3 kHz, the average hearing threshold is 41.25 dB in the initial record and 25 dB one month after treatment. The Patient-Personalized Seigel’s criteria could be better than the traditional method of capturing patient-specific hearing damage. A patient classified as recovered under conventional Siegel’s criteria might be deemed non-recovered when evaluated with the patient-specific frequency range. These findings emphasize the need for personalized assessments to accurately determine the prognosis of patients with ISSHL.

### 5.2. The Distinct Characteristics of this Study

The novelties of this study include (1) applying Patient-Personalized Seigel’s criteria to capture individual variability in hearing damage, (2) conducting statistical tests to determine the difference between traditional Seigel’s criteria and Patient-Personalized Seigel’s criteria, (3) eliminating detailed hearing threshold features to mitigate the model’s high reliance on hearing threshold features. Based on the result of these three keys, we emphasize the three main strengths of our research.

Patient-Personalized Seigel’s criteria not only assess recovery focusing on patient-specific hearing impairment but may also assess recovery with no significant difference from traditional Seigel’s criteria. Significant differences in recovery assessments could cause confusion for clinicians using traditional Seigel’s criteria, thus affecting their willingness to accept the newly applied Seigel’s criteria. There is one comparable study that developed an ISSHL prognostic model considering affected frequencies [8]. In this study, it could not be confirmed whether there is a significant difference in the recovery assessment result between affected criteria and traditional criteria. But, in our study, we confirmed a distribution shift in the recovery assessment when changing Seigel’s criteria and conducted a chi-square test to investigate the statistical significance of the shift. As a result, the chi-square value was about 2.61 and the *p*-value was 0.106, indicating no statistical significance in the difference between the two Seigel’s criteria.

By eliminating the detailed hearing threshold features, the ISSHL prognosis model could avoid issues related to the inaccurate measurement of detailed PTA records. In previous studies, detailed hearing level variables such as single hearing frequency have virtually the highest variable importance [5,6,7,8]. In our study, we included only the average hearing threshold as a hearing level variable in the dataset as follows: the average hearing thresholds of the affected and unaffected ears in the hearing-impaired frequency domains and its categorical variables. The average hearing thresholds of the affected and unaffected ears have the highest feature importance in our model. The models in previous studies could be vulnerable to the inaccurate hearing threshold of a single frequency because of its highest feature importance. If measuring the hearing threshold of certain frequencies is not possible or not accurately performed, there would be significant variance in the model’s prognosis prediction. But our model can avoid this potential limitation.

Also, despite utilizing a minimal set of hearing level variables, machine learning models succeeded in predicting the prognosis of ISSHL. Except for the decision tree and KNN, the AUROC scores were all over 0.80 in the test evaluation. The soft voting ensemble model was the best-performing model, with an AUROC of 0.864 (95% CI: 0.801–0.927). The soft voting ensemble model not only had the highest AUROC score but also a high rank in terms of balanced accuracy and highest precision in both validation and test evaluation. The soft-voting ensemble model addresses the weaknesses of the individual component models and reduces both the bias and variance of each component model [40].

### 5.3. Clinical Interpretation of the Soft-Voting Ensemble Model’s Prediction

We visualized the effect of 20 key variables of the soft voting ensemble model on recovery prediction using a SHAP summary plot. The average PTA value of the affected ear was found to have a negative impact on recovery prediction. Moderate hearing loss was the most frequent category in the recovery group, while profound hearing loss was the most frequent category in the non-recovery group regarding clinical characteristics. It is speculated that greater hearing damage implies more significant hearing loss, which, in turn, suggests substantial damage to hair cells, making recovery almost impossible [9,41]. The influence of the hearing level of the affected ear on recovery is consistent with numerous studies [6,41,42]. The high average PTA value in the unaffected ear also had a negative effect on recovery prediction in this study. Hearing impairment in the unaffected ear suggests a compromised state of the overall auditory system, making recovery less likely [43]. The influence of the extent of hearing in the unaffected ear on ISSHL recovery has been confirmed in multiple studies [43,44]. Additionally, although it was not possible to confirm the baseline hearing in the affected ear before the onset of ISSHL, assuming that hearing in both ears was symmetrical in most patients before the onset of ISSHL, hearing in the unaffected ear would have been similar to the baseline hearing in the affected ear. In such cases, the maximum hearing in the affected ear after treatment was similar to that in the unaffected ear, making hearing in the unaffected ear important for treatment outcomes. Binaural hearing is important for sound localization and distinguishing sound from noise; hence, the recovery of hearing in the unaffected ear up to the level of the unaffected hearing in the affected ear is crucial for daily life [45].

All five variables related to audiogram type were significant. The audiogram types with a positive effect on recovery prediction were flat, ascending, and U-shaped, whereas those with a negative effect were deaf and descending. These findings are consistent with those of a previous study that developed an ISSHL prognosis model [9]. We suggest that hearing recovery in the low- and mid-frequency regions is better than that in the high-frequency region [6,46,47]. This difference in recovery is attributed to variations in the metabolism and blood supply between the basal and apical cochlea [47]. Additionally, low-frequency sensorineural hearing loss can repeatedly occur as an early symptom of Meniere’s disease, which is characterized by endolymphatic hydrops. In this case, because of its fluctuating tendency, hearing recovery may appear to be more successful [48,49].

Systemic steroid therapy is known to be the most effective treatment for ISSHL. The spontaneous recovery rate of hearing in ISSHL is about 50% [47], but with systemic steroid therapy, approximately 80% of patients show hearing improvement [50]. Intratympanic dexamethasone injection (ITDI) is used as an additional treatment method for refractory ISSHL patients who do not respond to systemic steroids or concurrently with systemic steroids based on clinical judgment. Previous studies have indicated that the concurrent use of systemic steroids and ITDI is more effective in promoting recovery than the use of systemic steroids [51,52,53]. However, the SHAP summary plot in this study revealed a positive effect on prognosis prediction when only systemic steroids were administered, whereas other treatments, including ITDI, had negative effects. These contrasting results appear to be influenced by the severity of the initial hearing loss in the patients enrolled in this study. The group of patients who received systemic steroids as a single treatment had a lower level of hearing loss than the group of patients who received a combination of systemic steroids and ITDI. This may be due to the tendency for higher compliance with ITDI in patients with more severe hearing impairment, leading to patients with a higher initial PTA receiving additional ITDI treatment. Therefore, poor therapeutic outcomes observed in patients receiving a combination of systemic steroids and ITDI may be attributed to their poor initial hearing status.

The duration between onset and initial treatment, as well as the categorized duration between onset and initial ITDI treatment, negatively impacted the prediction of recovery as their extent increased. This negative impact is attributed to the timing of treatment. The longer the delay in treatment, the more challenging it is to recover from ISSHL. Previous studies demonstrated similar treatment delays [41,44,54,55,56].

Among blood test-related variables, BUN and lymphocyte count (%) were important for predicting recovery. A high BUN level was a negative predictor of recovery. We believe that an increase in the BUN level indicates a decrease in blood volume [57], which can lead to reduced blood flow to the inner ear. However, the correlation between BUN levels and hair cell damage has shown conflicting results across studies. Several studies on ISSHL have indicated that BUN is a negative prognostic factor for hearing [9,58]. Additionally, it has been shown that BUN levels are correlated with hearing loss in patients with chronic kidney disease [59,60]. On the other hand, other studies have found no correlation between BUN levels and hearing loss in CKD patients [61,62,63]. Therefore, further research is needed to clarify the correlation between BUN levels and cochlear damage. Higher levels of blood lymphocytes (%) have a positive impact on recovery prediction. It is believed that lymphoid cells play a role in regulating inflammatory responses and that regulatory T cells, which are a subtype of lymphoid cells, can help prevent arteriosclerosis [64]. Other statistical analyses indicate that lymphocytes (%) are significantly lower in non-recovered patient groups than in recovered patient groups [65].

In the health-related and democratic information categories, BMI, dizziness, tinnitus, age, weight, and diabetes are significant variables for recovery. Dizziness negatively affects recovery predictions. This is attributed to inflammatory reactions in the basal cochlea that extend into the semicircular canal, leading to dizziness [41]. Damage to the basal cochlea implies hearing loss in the high-frequency range, which contributes to a low recovery rate of ISSHL [6,46,47]. Previous studies have also highlighted the negative effects of tinnitus [41,42,43]. The onset of tinnitus can have variable effects on the prediction of recovery because recovery rates may differ based on the persistence time of tinnitus [66]. However, in our study, tinnitus onset had a positive effect on recovery prediction. This aligns with the findings of previous research [41,67]. The presence of tinnitus may indicate ongoing auditory cell function and potential for recovery, whereas the absence of tinnitus may indicate irreversible auditory cell damage [67]. Older age is presumed to have a negative effect on recovery prediction. We believe that older patients are prone to developing microangiopathy, which can lead to chronic inner ear damage due to insufficient oxygen supply [68], resulting in a negative impact of older age on recovery. The effect of older age on recovery prediction has been reported in previous studies [6,42,68,69]. Furthermore, high BMI and body weight negatively affect the prediction of recovery. This is likely due to increased body fat, which elevates blood fat levels and may hinder blood flow to the cochlea through microcirculation caused by increased blood viscosity [70]. Previous studies have highlighted the negative effect of BMI on recovery [70,71]. The presence of diabetes has a negative effect on prognosis, as reported in previous studies [72,73]. Microangiopathy of the cochlea in diabetes patients may lead to a low probability of ISSHL recovery [72,73]. Although the affected side and height are included in the list of the top 20 variables, they have little effect on prognostic prediction.

### 5.4. Limitations

This study has several limitations. The prediction of the model was restricted to one month post-treatment. Since the steroid-based treatment has long period effects, future models should aim to predict ISSHL recovery for longer than one month after treatment, such as three or six months after treatment. Moreover, the exclusion of patients because of missing PTA records could generate bias. The statistical significance of the recovery assessment shift should be conducted again with a larger dataset. Also, the application of Patient-Personalized Seigel’s criteria and model development with the elimination of detailed PTA features must be conducted using a larger dataset as well. Lastly, the high missing ratio not only in PTA features but also the other clinical features limits the completeness of the dataset, necessitating more data gathering and the development of more robust imputation strategies.

## 6. Conclusions

This retrospective study demonstrates that a soft voting classifier can effectively predict recovery from Idiopathic Sudden Sensorineural Hearing Loss (ISSHL) based on patient-specific hearing impairment frequency domains. Unlike traditional methods, this approach considers the individual variability in hearing loss, leading to a more accurate prognosis. We statistically tested the distribution shift of recovery assessment when changing from traditional Seigel’s criteria to Patient-Personalized Seigel’s criteria. In developing the model, we eliminated the detail hearing threshold parameters to mitigate the model’s high reliance on the PTA records. Factors that negatively impact prognosis were discovered based on the SHAP value, which included the following: average hearing threshold, dizziness, delay in treatment onset, and descending audiogram type. The distribution shift in the recovery assessment was not statistically significant based on the result of the chi-square test. Our study underscores the significance of the ISSHL prognostic model, which accounts for patient-specific hearing impairments. Further research is required to validate our findings in larger and more diverse patient populations. Further prospective studies are needed to investigate the impact of our machine learning models on clinical outcomes.

## Figures and Tables

**Table 1:** Variables in the utilized dataset.

	Continuous Variables	Binary/Categorical Variables
Democratic information	Age, height, weight	Gender (female)
Health records	Body mass index, extent of smoking(packs/year), systolic blood pressure, diastolic blood pressure	Smoking, smoking post-cessation status, 8 variables regarding presence of disease including (1) hypertension, (2) diabetes, (3) stroke, (4) dizziness, (5) tinnitus, (6) hyperlipidemia, (7) chronic kidney disease, and (8) myocardial infarction or angina.
Laboratory testing	Total cholesterol, low-density lipoprotein (LDL), triacylglycerol, hemoglobin, blood urea nitrogen (BUN), creatine (Cr), white blood cell count, neutrophil count, lymphocyte count, neutrophil–lymphocyte ratio, platelet count, prothrombin time, and activated partial thromboplastin time	None
Onset and Treatment	Duration between the onset of ISSHL and initial treatment, euration between the onset of ISSHL and ITDI treatment	Hospitalization, affected side (left), categorized variables of duration between the onset of ISSHL and initial ITDI treatment, onset month of ISSHL, length of the hearing-impaired frequency domain, three variables of steroid treatment type including systemic steroid, ITDI, and combined method of systemic steroid and ITDI
PTA records	The PTA average of the affected frequency domains in the affected and unaffected ears	Categorized variables of the PTA average of the affected frequency domains in the affected and unaffected ears, five variables of audiogram type regarding the initial PTA record including ascending, U-shaped, descending, flat, and deaf

PTA: pure tone audiometry, ISSHL: Idiopathic Sudden Sensorineural Hearing Loss, ITDI: initial intratympanic dexamethasone injection.

**Table 2:** Parameter settings for grid search by model and optimal parameters.

Model	Parameter	Settings	Optimal Set of Parameters
Logistic regression	C	(0.7, 1.0, 1.2)	1.0
Decision tree	ccp_alpha	(0.005, 0.01, 0.015, 0.02, 0.025)	0.02
Random Forest	n_estimators	(50, 100, 150)	100
	ccp_alpha	(0.01, 0.05, 0.1)	0.05
Support Vector Machine	C	(0.4, 0.6, 0.8)	0.6
	kernel	(“linear”)	“linear”
	degree	(2, 3)	2
AdaBoost	n_estimators	(20, 40, 60, 100)	20
	learning_rate	(0.6, 1.0, 1.4)	0.6
XGBoost	n_estimators	(50, 100, 200)	50
	learning_rate	(0.6, 0.8, 1.0, 1.2)	0.6
	reg_alpha	(0.4, 0.8, 1.2, 1.6)	0.8
	reg_lambda	(1.4, 1.8, 2.2, 2.6)	2.6
	gamma	(0.6, 1.0, 1.4, 1.8)	0.6
LGBM	n_estimators	(25, 50, 100)	25
	learning_rate	(0.2, 0.4, 0.6, 0.8, 1.0)	0.2
	reg_alpha	(0.8, 1.2, 1.6, 2.0)	2.0
	reg_lambda	(0.8, 1.2, 1.6, 2.0)	2.0
K-Nearest Neighbors	n_neighbors	(5, 10, 15, 20, 25)	25
	weights	(“uniform”, “distance”)	“distance”
Soft-Voting ensemble	none	none	none

**Table 3:** Comparison of recovery and non-recovery patients with a variable that has a statistically significant difference.

Variable	Non-Recovery (*n* = 361)	Recovery (*n* = 220)	Total (*n* = 581)	*p*-Value
Continuous variables, median (Q_1_, Q_3_)				
Age, year	55.00 (47.00, 64.00)	48.00 (38.25, 57.00)	52.00 (43.00, 60.00)	<0.001
Triacylglycerol, mg/dL	99.00 (66.50, 148.00)	82.00 (56.00, 132.00)	93.00 (61.00, 142.50)	0.006
Missing values, No. (%)	156 (43.21)	77 (35.00)	233 (40.10)	
Blood urea nitrogen, mg/dL	15.20 (12.40, 19.58)	13.60 (11.50, 16.00)	14.50 (11.90, 18.30)	<0.001
Missing values, No. (%)	49 (13.57)	21 (9.55)	70 (12.05)	
Creatinine, mg/dL	0.88 (0.71, 1.04)	0.83 (0.70, 0.98)	0.86 (0.70, 1.02)	0.025
Missing values, No. (%)	41 (11.36)	16 (7.27)	57 (9.81)	
Duration time between onset and ITDI treatment, day	6.00 (3.00, 16.00)	5.00 (2.00, 8.50)	6.00 (2.00, 13.50)	0.003
Missing values, No. (%)	137 (37.95)	95 (43.18)	232 (39.93)	
PTA average of affected frequency range (AE), dB	75.63 (56.77, 98.44)	61.25 (48.33, 77.34)	69.38 (51.25, 90.00)	<0.001
PTA average of affected frequency range (UAE), dB	23.13 (15.00, 36.25)	16.88 (10.83, 23.59)	20.00 (13.00, 30.73)	<0.001
Categorical variables, No. (%)				
Hypertension	131 (36.29)	46 (20.91)	177 (30.46)	<0.001
Missing values, No. (%)	2 (0.6)	3 (1.4)	5 (0.9)	
Diabetes	113 (31.30)	47 (21.36)	160 (27.54)	0.01
Missing values, No. (%)	2 (0.6)	2 (0.9)	4 (0.7)	
Myocardial infarction or angina	21 (5.82)	3 (1.4)	24 (4.13)	0.009
Missing values, No. (%)	2 (0.6)	3 (1.4)	5 (0.9)	
Dizziness	152 (42.11)	39 (17.73)	191 (32.87)	<0.001
Missing values, No. (%)	1 (0.3)	2 (0.9)	3 (0.5)	
Tinnitus	230 (63.71)	164 (74.55)	394 (67.81)	0.006
Missing values, No. (%)	1 (0.3)	1 (0.5)	2 (0.3)	
Category of time between onset and ITDI treatment				0.005
1 (0–3 days from onset)	72 (19.94)	51 (23.18)	123 (21.17)	
2 (4–7 days from onset)	51 (14.13)	40 (18.18)	91 (15.66)	
3 (8–12 days from onset)	28 (7.76)	14 (6.36)	42 (7.23)	
4 (13~ days from onset)	73 (20.22)	20 (9.09)	93 (16.01)	
Missing values, No. (%)	137 (37.95)	95 (43.18)	232 (39.93)	
Categorized severity level of PTA average (AE)				<0.001
1 (Mild: 20 dB to 40 dB)	10 (2.77)	14 (6.36)	24 (4.13)	
2 (Moderate: 40 dB to 60 dB)	87 (24.10)	90 (40.91)	177 (30.46)	
3 (Severe: 60 dB to 80 dB)	100 (27.70)	68 (30.91)	168 (28.92)	
4 (Profound: 80 dB to 100 dB)	119 (32.96)	46 (20.91)	165 (28.40)	
5 (Deaf: ≥100 dB)	45 (12.47)	2 (0.91)	47 (8.09)	
Categorized severity level of PTA average (UAE)				<0.001
1 (Mild: 20 dB to 40 dB)	282 (78.12)	207 (94.09)	489 (84.17)	
2 (Moderate: 40 dB to 60 dB)	45 (12.47)	5 (2.27)	50 (8.61)	
3 (Severe: 60 dB to 80 dB)	18 (4.99)	5 (2.27)	23 (3.96)	
4 (Profound: 80 dB to 100 dB)	14 (3.88)	2 (0.91)	16 (2.75)	
5 (Deaf: ≥100 dB)	2 (0.55)	1 (0.45)	3 (0.52)	
Audiogram type—ascending	35 (9.69)	47 (21.36)	82 (14.11)	<0.001
Audiogram type—U-shaped	17 (4.71)	28 (12.72)	45 (7.75)	<0.001
Audiogram type—descending	119 (32.96)	53 (24.09)	172 (29.60)	0.023
Audiogram type—flat	81 (22.44)	83 (37.73)	164 (28.22)	<0.001
Audiogram type—deaf	109 (30.19)	9 (4.09)	118 (20.31)	<0.001
Length of affected frequency range				0.001
3	21 (5.82)	24 (10.91)	45 (7.75)	
4	18 (4.99)	21 (9.55)	39 (6.71)	
5	20 (5.54)	11 (5.00)	31 (5.34)	
6	17 (4.71)	17 (7.73)	34 (5.85)	
7	13 (3.60)	16 (7.27)	29 (4.99)	
8	272 (75.34)	131 (59.55)	403 (69.36)	

All variables in this table show statistically significant differences between the two groups at a significance level of 0.05. For continuous variables, the Shapiro–Wilk test assessing the normality of the distribution of continuous variables, an independent sample t-test (normally distributed), or a Mann–Whitney U test (not normally distributed) was conducted. For categorical variables, chi-square or Fisher’s exact test was conducted. ITDI: initial intratympanic dexamethasone injection, PTA: pure tone audiometry, dB: decibel, AE: affected ear, UAE: unaffected ear.

**Table 4:** Validation performance of the machine learning models.

Metrics	Machine Learning Models								
	LR	DT	RF	SVM	ADA	XGB	LGBM	KNN	SVC
BACC	0.685	0.677	0.684	0.676	0.664	0.651	0.675	0.624	0.686
Recall	0.695	0.659	0.673	0.705	0.548	0.514	0.618	0.434	0.597
Precision	0.569	0.577	0.587	0.552	0.610	0.598	0.587	0.591	0.620
F1	0.624	0.609	0.618	0.618	0.573	0.548	0.599	0.497	0.605
AUROC	0.767	0.707	0.766	0.761	0.748	0.715	0.743	0.719	0.775
AUROC 95% CI	0.646–0.878	0.596–0.816	0.644–0.880	0.636–0.877	0.625–0.865	0.581–0.846	0.613–0.867	0.595–0.835	0.659–0.887

BACC: balanced accuracy, CI: confidence interval, AUROC: Area Under the Receiver Operating Characteristic Curve, LR: logistic regression, DT: decision tree, RF: Random Forest, SVM: Support Vector Machine, ADA: Adaptive Boosting (AdaBoost), XGB: Extreme Gradient Boosting (XGBoost), LGBM: Light Gradient Boost Model, KNN: K-Nearest Neighbor, SVC: Soft Voting Classifier.

**Table 5:** Test Evaluation Performance of the Machine Learning Models.

Metrics	Machine Learning Models								
	LR	DT	RF	SVM	ADA	XGB	LGBM	KNN	SVC
BACC	0.797	0.706	0.722	0.788	0.745	0.722	0.747	0.611	0.772
Recall	0.841	0.727	0.909	0.864	0.682	0.636	0.727	0.455	0.750
Precision	0.673	0.582	0.541	0.644	0.682	0.667	0.653	0.541	0.688
F1	0.747	0.646	0.678	0.738	0.682	0.651	0.688	0.494	0.717
AUROC	0.850	0.752	0.803	0.861	0.858	0.832	0.847	0.734	0.864
AUROC 95% CI	0.781–0.918	0.671–0.832	0.734–0.872	0.792–0.929	0.797–0.919	0.756–0.907	0.780–0.913	0.639–0.829	0.801–0.927

## Data Availability

The dataset used in this study is not publicly available. However, the data in this study can be provided upon reasonable request to the corresponding author.

## Supplementary Materials

The following supporting information can be downloaded from https://www.mdpi.com/article/10.3390/diagnostics14121296/s1, Table S1: Python software packages, Table S2: Detailed comparison between recovery and non-recovery.
