# Supplementary material for: Predicting Sudden Sensorineural Hearing Loss Recovery with Patient-Personalized Seigel’s Criteria Using Machine Learning
Source: Diagnostics (Basel). 2024 Jun 19;14(12):1296. doi: 10.3390/diagnostics14121296 (PMC11202901; doi:10.3390/diagnostics14121296)
Supplement: Supplementary file 1 [file diagnostics-14-01296-s001.zip › diagnostics-2987221-supplementary.pdf]

Supplementary Materials

Table S1. Python Software packages

Python packages	Version
numpy	1.26.1
pandas	1.4.2
Pyplot	3.5.1
sklearn	1.2.2
xgboost	1.7.2
lightgbm	3.3.4
shap	0.41.0

Table S2. Detailed comparison between recovery and non-recovery

Variable	Non-recovery (n=361)	Recovery (n=220)	Total (n=581)	P-value
**Continuous variables, median (IQR)**				
Age, year	55.00 (47.00-64.00)	48.00 (38.25-57.00)	52.00 (43.00-60.00)	<0.001
Height, cm	162.70 (156.00, 169.05)	163.00 (156.00, 169.43)	162.85 (156.00-169.20)	0.955
Weight, kg	65.25 (56.41-72.70)	63.00 (55.18-73.05)	64.40 (56.00-72.70)	0.175
Body mass index	24.60 (22.47-26.46)	24.30 (21.41-26.29)	24.51 (22.07-26.41)	0.156
Amount of smoking, pack per year	0.00 (0.00-0.00)	0 (0.00-0.00)	0.00 (0.00-0.00)	0.304
Systolic blood pressure, mmHg	120.00 (110.00-130.00)	120.00 (110.00-130.00)	120.00 (110.00-130.00)	0.229
Diastolic blood pressure, mmHg	80.00 (70.00-80.00)	80.00 (70.00-80.00)	80.00 (70.00-80.00)	0.731
Total cholesterol, mg/dL	178.00 (151.75-215.25)	179.00 (153.00-206.25)	179.00 (153.00-212.00)	0.963
LDL, mg/dL	102.00 (74.00-138.00)	102.50 (75.00-125.75)	102.00 (75.00-130.00)	0.678
Triacylglycerol, mg/dL	99.00 (66.50-148.00)	82.00 (56.00-132.00)	93.00 (61.00-142.50)	0.006
Hemoglobin, g/dL	13.60 (12.50-14.70)	13.60 (12.50-14.70)	13.60 (12.50-14.70)	0.948
Prothrombin time, second	11.20 (10.60-11.90)	11.10 (10.50-12.00)	11.20 (10.50-11.90)	0.447
Activated partial thromboplastin time, second	29.20 (26.50-31.90)	28.90 (27.13-32.30)	29.10 (26.80-32.03)	0.661
Blood urea nitrogen, mg/dL	15.20 (12.40-19.58)	13.60 (11.50-16.00)	14.50 (11.90-18.30)	<0.001
Creatinine, mg/dL	0.88 (0.71-1.04)	0.83 (0.70-0.98)	0.86 (0.70-1.02)	0.025
White blood cell count, $10^3/\mu L$	7.70 (6.19-10.28)	7.81 (6.12-10.30)	7.77 (6.15-10.29)	0.522
Neutrophil percent, %	68.60 (56.10-83.40)	66.80 (56.00-83.90)	68.30 (56.08-83.53)	0.789
Lymphocyte percent, %	22.80 (12.40, 32.40)	24.30 (11.90-34.00)	23.45 (12.20-33.20)	0.686
Neutrophil to lymphocyte ratio	2.98 (1.72-6.65)	2.79 (1.67-6.88)	2.94 (1.70-6.79)	0.705
Platelet count, $10^3/\mu L$	225.00 (191.00-278.00)	227.00 (204.00-282.00)	227.00 (197.75-281.00)	0.093

Duration time between onset and treatment, day	3.00 (1.00-7.00)	3.00 (1.00-4.75)	3.00 (1.00-6.00)	0.175
Duration time between onset and ITDI treatment, day	6.00 (3.00-16.00)	5.00 (2.00-8.50)	6.00 (2.00-13.50)	0.003
PTA average of affected frequency range (AE), dB	75.63 (56.77-98.44)	61.25 (48.33-77.34)	69.38 (51.25-90.00)	<0.001
PTA average of affected frequency range (UAE), dB	23.13 (15.00-36.25)	16.88 (10.83-23.59)	20.00 (13.00-30.73)	<0.001
**Categorical variables, No. (%)**				
Side (Left)	190 (52.63)	116 (52.73)	306 (52.67)	0.982
Sex (Female)	176 (48.75)	121 (55.00)	297 (51.12)	0.144
Hospitalization	318 (88.09)	190 (86.36)	508 (87.44)	0.543
Hypertension	131 (36.49)	46 (21.20)	177 (30.73)	<0.001
Diabetes	113 (31.48)	47 (21.56)	160 (27.73)	0.010
Hyperlipidemia	30 (8.36)	21 (9.68)	51 (8.85)	0.589
Stroke	6 (1.67)	3 (1.38)	9 (1.56)	0.787
Chronic Kidney Disease	15 (4.18)	3 (1.38)	18 (3.13)	0.062
Myocardial Infarction or Angina	21 (5.85)	3 (1.38)	24 (4.17)	0.009
Onset month of ISSHL				0.916
1(January)	22(6.13)	14(6.36)	36 (6.22)	
2(February)	41(11.42)	26(11.82)	67 (11.57)	
3(March)	33(9.19)	25(11.36)	58 (10.02)	
4(April)	23(6.41)	20(9.09)	43 (7.43)	
5(May)	30(8.36)	16(7.27)	46 (7.94)	
6(June)	31(8.64)	22(10.00)	53 (9.15)	
7(July)	33(9.19)	16(7.27)	49 (8.46)	
8(August)	34(9.47)	15(6.82)	49 (8.46)	
9(September)	26(7.24)	13(5.91)	39 (6.74)	
10(October)	26(7.24)	17(7.73)	43 (7.43)	
11(November)	20(5.57)	15(6.82)	35 (6.04)	
12(December)	40(11.14)	21(9.55)	61 (10.54)	
Dizziness	152 (42.22)	39 (17.89)	191 (33.04)	<0.001
Tinnitus	230 (63.89)	164 (74.89)	394 (68.05)	0.006
Category of time between onset and ITDI treatment				0.005
1 (0~3 days from onset)	72 (32.14)	51 (40.80)	123 (35.24)	
2 (4~7 days from onset)	51 (22.77)	40 (32.00)	91 (26.07)	
3 (8~12 days from onset)	28 (12.50)	14 (11.20)	42 (12.03)	
4 (13~ days from onset)	73 (32.59)	20 (16.00)	93 (26.65)	
Categorized severity level of PTA average (AE)				<0.001
1 (Mild: 20≤PTA average<40)	10 (2.77)	14 (6.36)	24 (4.13)	
2 (Moderate: 40≤PTA average<59)	87 (24.10)	90 (40.91)	177 (30.46)	
3 (Severe: 60≤PTA average<79)	100 (27.70)	68 (30.91)	168 (28.92)	
4 (Profound: 80≤PTA average<100)	119 (32.96)	46 (20.91)	165 (28.40)	
5 (Deaf: 100≤PTA average)	45 (12.47)	2 (0.91)	47 (8.09)	
Categorized severity level of PTA average (UAE)				<0.001
1 (Mild: 20≤PTA average<40)	282 (78.12)	207 (94.09)	489 (84.17)	
2 (Moderate: 40≤PTA average<59)	45 (12.47)	5 (2.27)	50 (8.61)	
3 (Severe: 60≤PTA average<79)	18 (4.99)	5 (2.27)	23 (3.96)	
4 (Profound: 80≤PTA average<100)	14 (3.88)	2 (0.91)	16 (2.75)	

5 (Deaf: 100≤PTA average)	2 (0.55)	1 (0.45)	3 (0.52)	
Smoking	49 (13.57)	32 (14.55)	81 (13.94)	0.743
History of smoking	12 (3.32)	12 (5.45)	24 (4.13)	0.211
Systemic steroid - oral or IV injection	134 (37.12)	94 (42.73)	228 (39.24)	0.179
Systemic steroid - oral or IV injection + ITDI	218 (60.39)	123 (55.91)	341 (58.69)	0.288
Systemic steroid - ITDI only	9 (2.49)	3 (1.36)	12 (2.07)	0.549
Audiogram type-Ascending	35 (9.70)	47 (21.36)	82 (14.11)	<0.001
Audiogram type-U shape	17 (4.71)	28 (12.73)	45 (7.75)	<0.001
Audiogram type-Descending	119 (32.96)	53 (24.09)	172 (29.60)	0.023
Audiogram type-Flat	81 (22.44)	83 (37.73)	164 (28.23)	<0.001
Audiogram type-Deaf	109 (30.19)	9 (4.09)	118 (20.31)	<0.001
Length of consecutive affected frequency range				0.001
3	21 (5.82)	24 (10.91)	45 (7.75)	
4	18 (4.99)	21 (9.55)	39 (6.71)	
5	20 (5.54)	11 (5.00)	31 (5.34)	
6	17 (4.71)	17 (7.73)	34 (5.85)	
7	13 (3.60)	16 (7.27)	29 (4.99)	
8	272 (75.35)	131 (59.55)	403 (69.36)	
